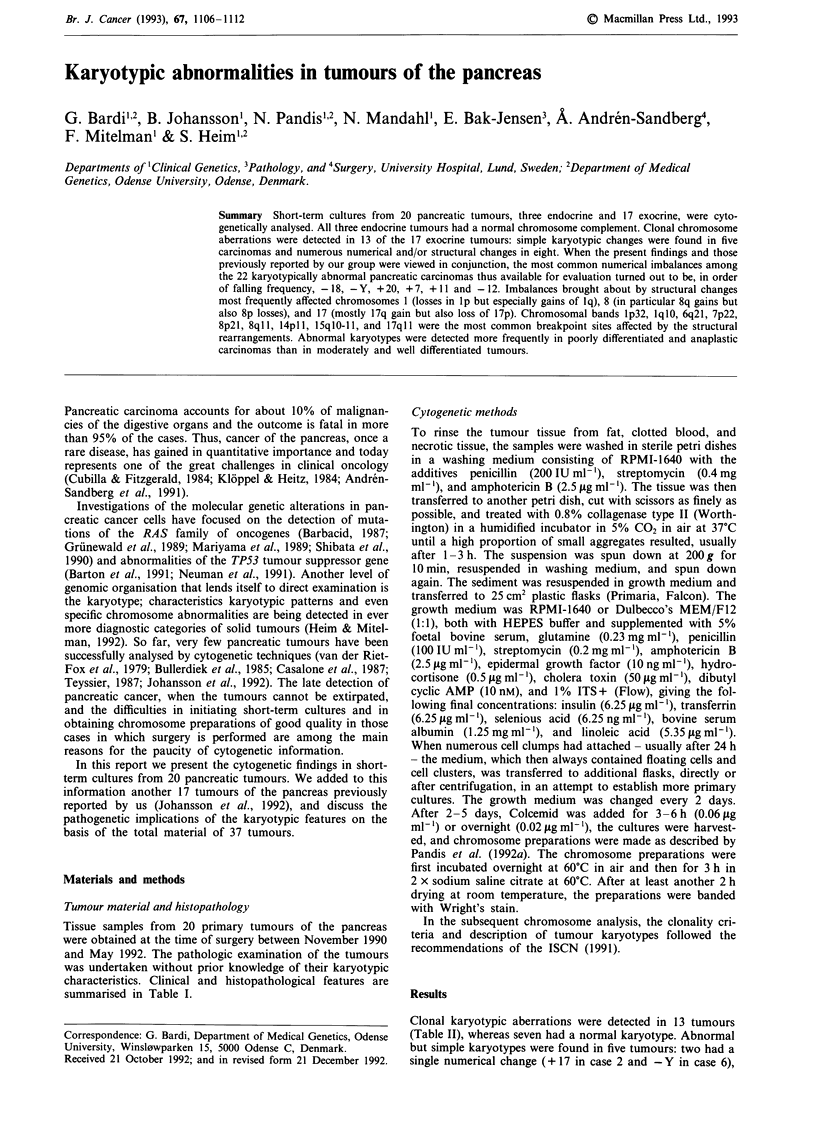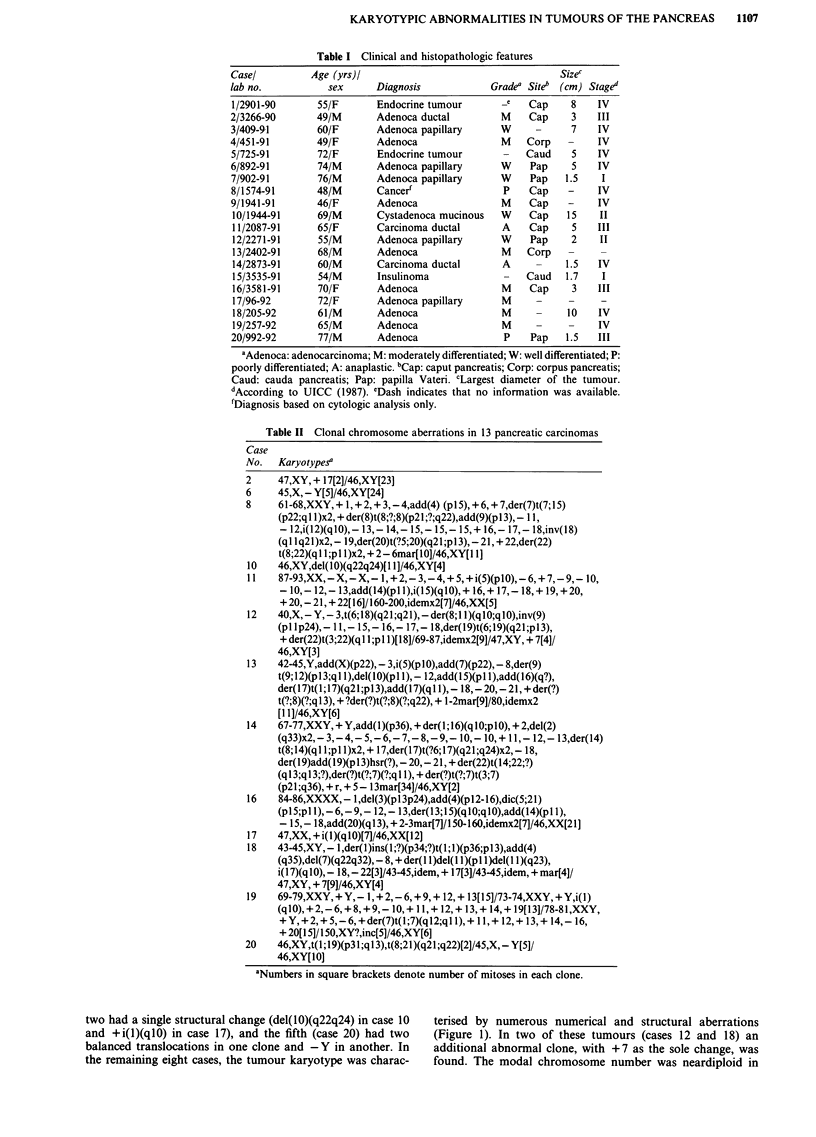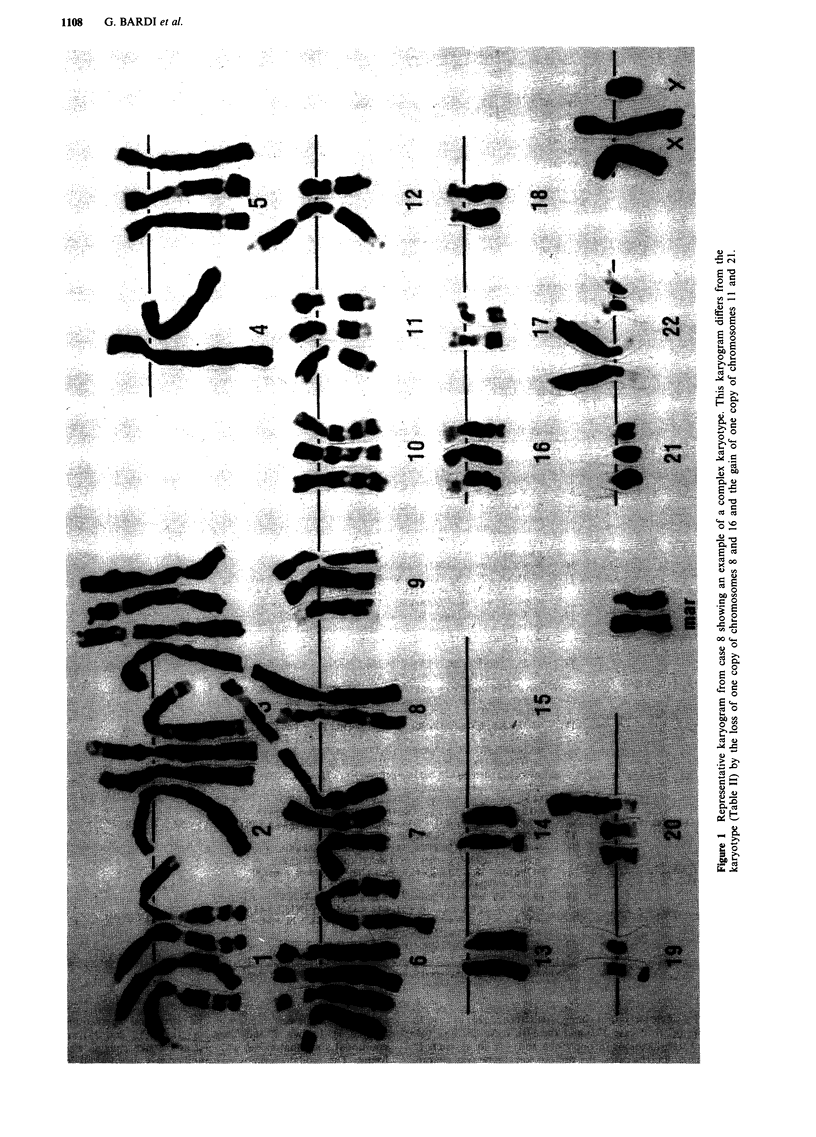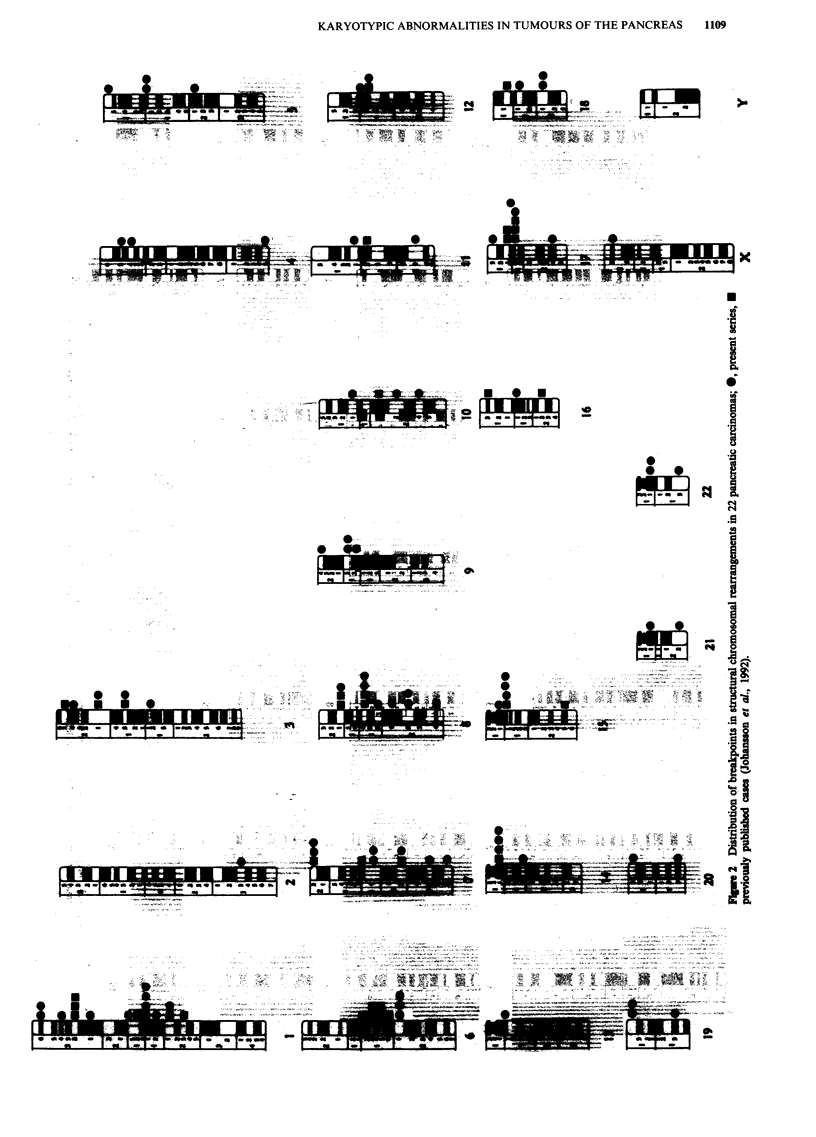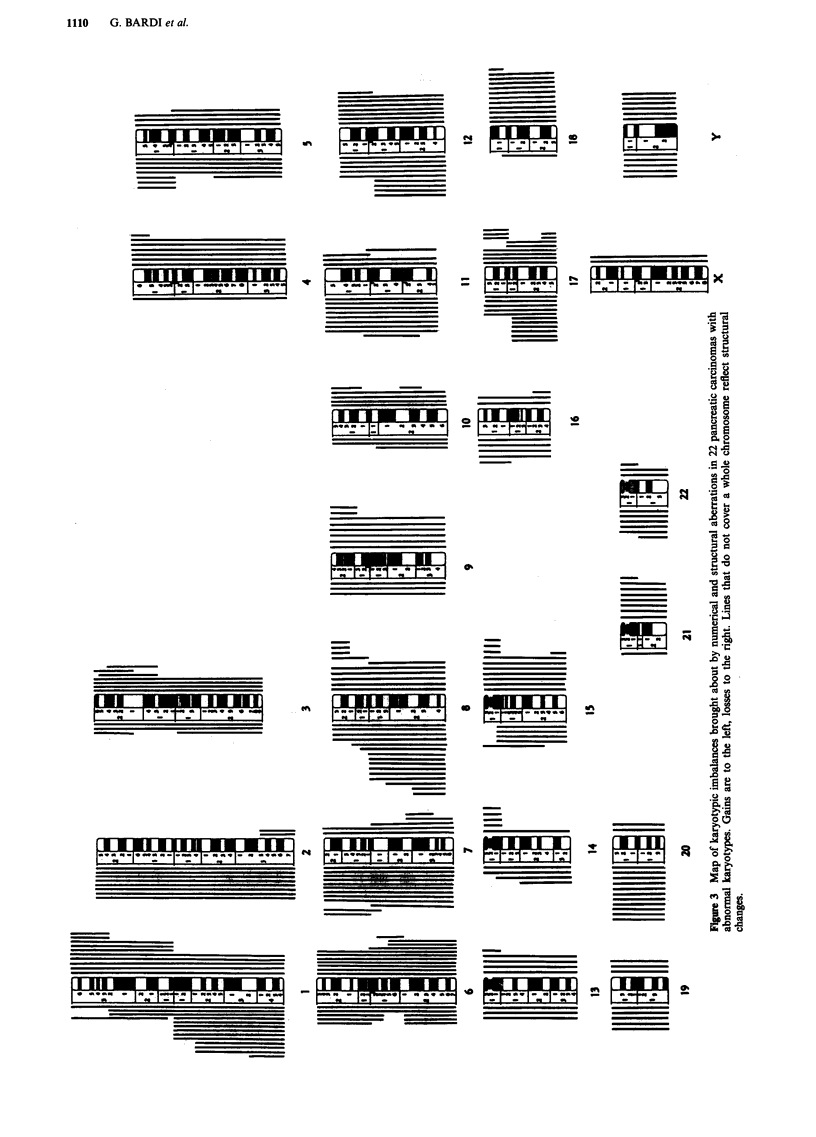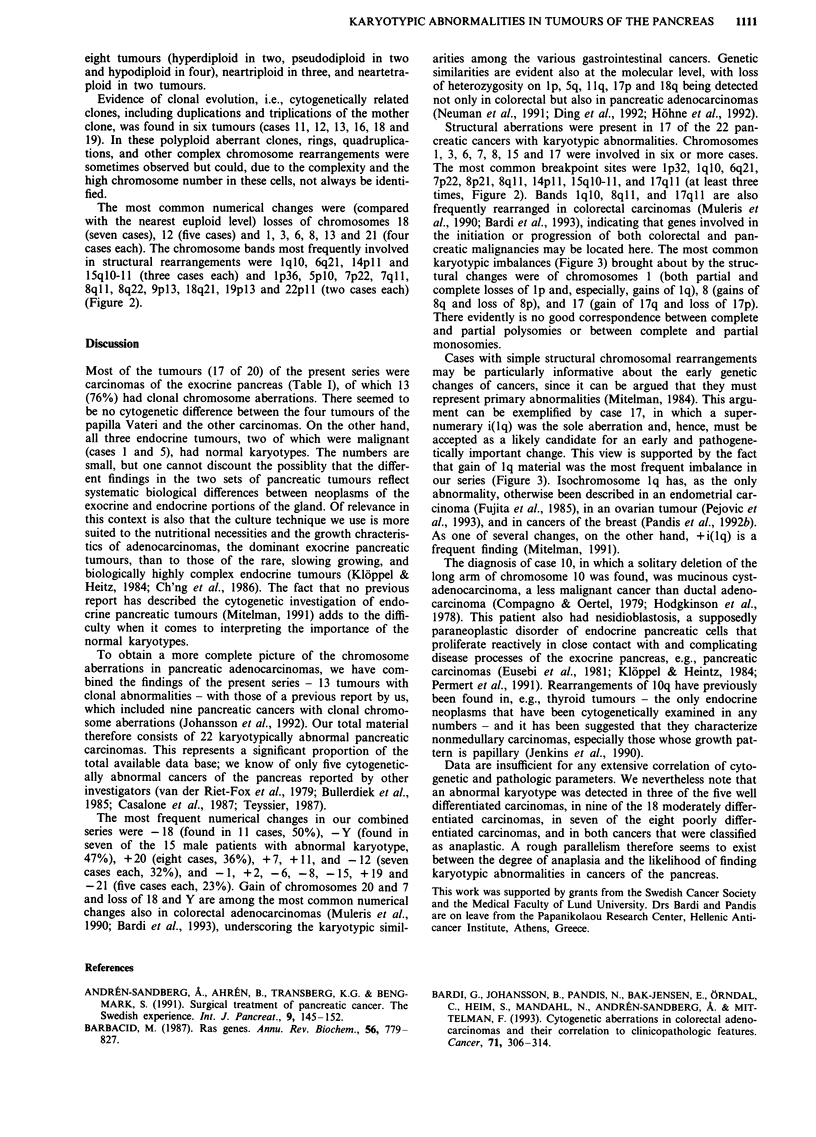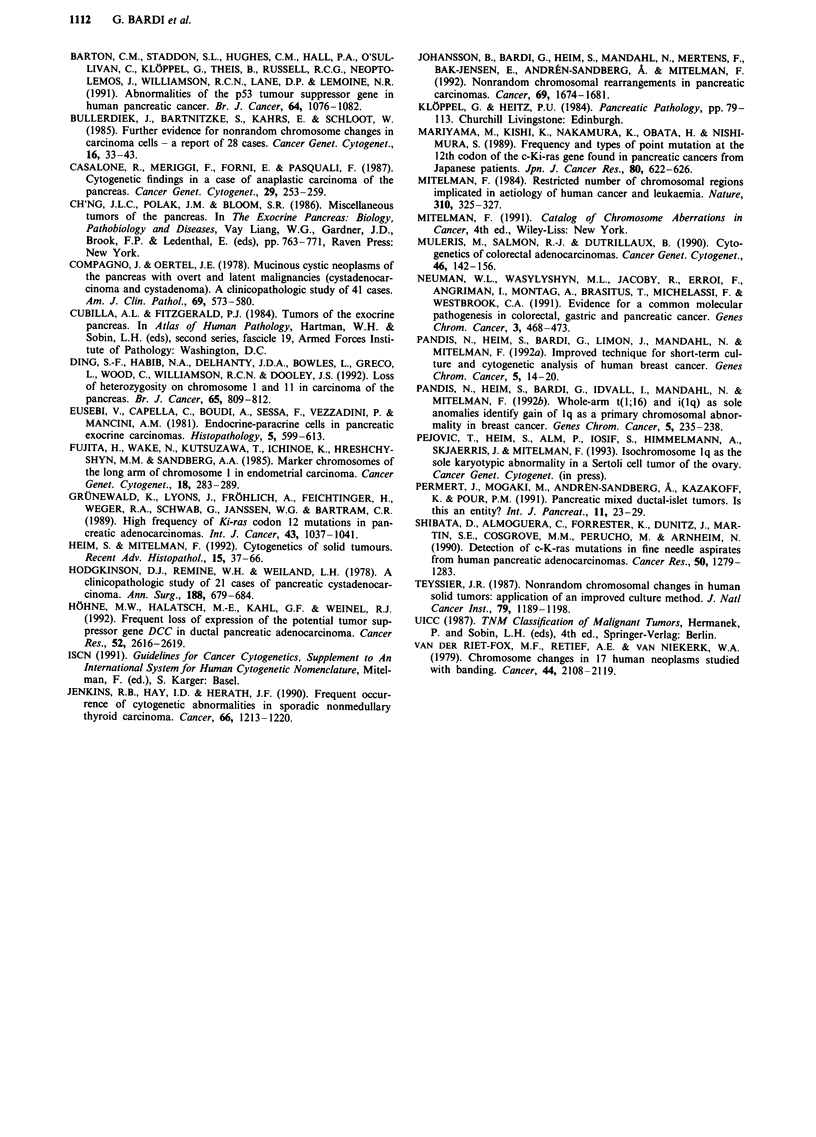# Karyotypic abnormalities in tumours of the pancreas.

**DOI:** 10.1038/bjc.1993.203

**Published:** 1993-05

**Authors:** G. Bardi, B. Johansson, N. Pandis, N. Mandahl, E. Bak-Jensen, A. Andrén-Sandberg, F. Mitelman, S. Heim

**Affiliations:** Department of Clinical Genetics, University Hospital, Lund, Sweden.

## Abstract

**Images:**


*Br. J. Cancer* (1993), **67**, 1106–1112 © Macmillan Press Ltd., 1993

### Karyotypic abnormalities in tumours of the pancreas

G. Bardi[1,2], B. Johansson[1], N. Pandis[1,2], N. Mandahl[1], E. Bak-Jensen[3], Å. Andrén-Sandberg[4], F. Mitelman[1] & S. Heim[1,2]

*Departments of [1]Clinical Genetics, [3]Pathology, and [4]Surgery, University Hospital, Lund, Sweden; [2]Department of Medical Genetics, Odense University, Odense, Denmark.*

**Summary** Short-term cultures from 20 pancreatic tumours, three endocrine and 17 exocrine, were cytogenetically analysed. All three endocrine tumours had a normal chromosome complement. Clonal chromosome aberrations were detected in 13 of the 17 exocrine tumours: simple karyotypic changes were found in five carcinomas and numerous numerical and/or structural changes in eight. When the present findings and those previously reported by our group were viewed in conjunction, the most common numerical imbalances among the 22 karyotypically abnormal pancreatic carcinomas thus available for evaluation turned out to be, in order of falling frequency, −18, −Y, +20, +7, +11 and −12. Imbalances brought about by structural changes most frequently affected chromosomes 1 (losses in 1p but especially gains of 1q), 8 (in particular 8q gains but also 8p losses), and 17 (mostly 17q gain but also loss of 17p). Chromosomal bands 1p32, 1q10, 6q21, 7p22, 8p21, 8q11, 14p11, 15q10-11, and 17q11 were the most common breakpoint sites affected by the structural rearrangements. Abnormal karyotypes were detected more frequently in poorly differentiated and anaplastic carcinomas than in moderately and well differentiated tumours.

Pancreatic carcinoma accounts for about 10% of malignancies of the digestive organs and the outcome is fatal in more than 95% of the cases. Thus, cancer of the pancreas, once a rare disease, has gained in quantitative importance and today represents one of the great challenges in clinical oncology (Cubilla & Fitzgerald, 1984; Klöppel & Heitz, 1984; Andrén-Sandberg *et al.*, 1991).

Investigations of the molecular genetic alterations in pancreatic cancer cells have focused on the detection of mutations of the *RAS* family of oncogenes (Barbacid, 1987; Grünewald *et al.*, 1989; Mariyama *et al.*, 1989; Shibata *et al.*, 1990) and abnormalities of the *TP53* tumour suppressor gene (Barton *et al.*, 1991; Neuman *et al.*, 1991). Another level of genomic organisation that lends itself to direct examination is the karyotype; characteristics karyotypic patterns and even specific chromosome abnormalities are being detected in ever more diagnostic categories of solid tumours (Heim & Mitelman, 1992). So far, very few pancreatic tumours have been successfully analysed by cytogenetic techniques (van der Riet-Fox *et al.*, 1979; Bullerdiek *et al.*, 1985; Casalone *et al.*, 1987; Teyssier, 1987; Johansson *et al.*, 1992). The late detection of pancreatic cancer, when the tumours cannot be extirpated, and the difficulties in initiating short-term cultures and in obtaining chromosome preparations of good quality in those cases in which surgery is performed are among the main reasons for the paucity of cytogenetic information.

In this report we present the cytogenetic findings in short-term cultures from 20 pancreatic tumours. We added to this information another 17 tumours of the pancreas previously reported by us (Johansson *et al.*, 1992), and discuss the pathogenetic implications of the karyotypic features on the basis of the total material of 37 tumours.

### Materials and methods

### *Tumour material and histopathology*

Tissue samples from 20 primary tumours of the pancreas were obtained at the time of surgery between November 1990 and May 1992. The pathologic examination of the tumours was undertaken without prior knowledge of their karyotypic characteristics. Clinical and histopathological features are summarised in Table I.

Correspondence: G. Bardi, Department of Medical Genetics, Odense University, Winsløwparken 15, 5000 Odense C, Denmark.
Received 21 October 1992; and in revised form 21 December 1992.

### *Cytogenetic methods*

To rinse the tumour tissue from fat, clotted blood, and necrotic tissue, the samples were washed in sterile petri dishes in a washing medium consisting of RPMI-1640 with the additives penicillin (200 IU ml$^{-1}$), streptomycin (0.4 mg ml$^{-1}$), and amphotericin B (2.5 μg ml$^{-1}$). The tissue was then transferred to another petri dish, cut with scissors as finely as possible, and treated with 0.8% collagenase type II (Worthington) in a humidified incubator in 5% $CO_2$ in air at 37°C until a high proportion of small aggregates resulted, usually after 1–3 h. The suspension was spun down at 200 *g* for 10 min, resuspended in washing medium, and spun down again. The sediment was resuspended in growth medium and transferred to 25 cm$^2$ plastic flasks (Primaria, Falcon). The growth medium was RPMI-1640 or Dulbecco's MEM/F12 (1:1), both with HEPES buffer and supplemented with 5% foetal bovine serum, glutamine (0.23 mg ml$^{-1}$), penicillin (100 IU ml$^{-1}$), streptomycin (0.2 mg ml$^{-1}$), amphotericin B (2.5 μg ml$^{-1}$), epidermal growth factor (10 ng ml$^{-1}$), hydrocortisone (0.5 μg ml$^{-1}$), cholera toxin (50 μg ml$^{-1}$), dibutyl cyclic AMP (10 nM), and 1% ITS+ (Flow), giving the following final concentrations: insulin (6.25 μg ml$^{-1}$), transferrin (6.25 μg ml$^{-1}$), selenious acid (6.25 ng ml$^{-1}$), bovine serum albumin (1.25 mg ml$^{-1}$), and linoleic acid (5.35 μg ml$^{-1}$). When numerous cell clumps had attached – usually after 24 h – the medium, which then always contained floating cells and cell clusters, was transferred to additional flasks, directly or after centrifugation, in an attempt to establish more primary cultures. The growth medium was changed every 2 days. After 2–5 days, Colcemid was added for 3–6 h (0.06 μg ml$^{-1}$) or overnight (0.02 μg ml$^{-1}$), the cultures were harvested, and chromosome preparations were made as described by Pandis *et al.* (1992*a*). The chromosome preparations were first incubated overnight at 60°C in air and then for 3 h in 2 × sodium saline citrate at 60°C. After at least another 2 h drying at room temperature, the preparations were banded with Wright's stain.

In the subsequent chromosome analysis, the clonality criteria and description of tumour karyotypes followed the recommendations of the ISCN (1991).

### Results

Clonal karyotypic aberrations were detected in 13 tumours (Table II), whereas seven had a normal karyotype. Abnormal but simple karyotypes were found in five tumours: two had a single numerical change (+17 in case 2 and −Y in case 6),

**Table I** Clinical and histopathologic features

| *Case/ lab no.* | *Age (yrs)/ sex* | *Diagnosis* | *Grade*[a] | *Site*[b] | *Size*[c] *(cm)* | *Stage*[d] |
|---|---|---|---|---|---|---|
| 1/2901-90 | 55/F | Endocrine tumour | –[e] | Cap | 8 | IV |
| 2/3266-90 | 49/M | Adenoca ductal | M | Cap | 3 | III |
| 3/409-91 | 60/F | Adenoca papillary | W | – | 7 | IV |
| 4/451-91 | 49/F | Adenoca | M | Corp | – | IV |
| 5/725-91 | 72/F | Endocrine tumour | – | Caud | 5 | IV |
| 6/892-91 | 74/M | Adenoca papillary | W | Pap | 5 | IV |
| 7/902-91 | 76/M | Adenoca papillary | W | Pap | 1.5 | I |
| 8/1574-91 | 48/M | Cancer[f] | P | Cap | – | IV |
| 9/1941-91 | 46/F | Adenoca | M | Cap | – | IV |
| 10/1944-91 | 69/M | Cystadenoca mucinous | W | Cap | 15 | II |
| 11/2087-91 | 65/F | Carcinoma ductal | A | Cap | 5 | III |
| 12/2271-91 | 55/M | Adenoca papillary | W | Pap | 2 | II |
| 13/2402-91 | 68/M | Adenoca | M | Corp | – | – |
| 14/2873-91 | 60/M | Carcinoma ductal | A | – | 1.5 | IV |
| 15/3535-91 | 54/M | Insulinoma | – | Caud | 1.7 | I |
| 16/3581-91 | 70/F | Adenoca | M | Cap | 3 | III |
| 17/96-92 | 72/F | Adenoca papillary | M | – | – | – |
| 18/205-92 | 61/M | Adenoca | M | – | 10 | IV |
| 19/257-92 | 65/M | Adenoca | M | – | – | IV |
| 20/992-92 | 77/M | Adenoca | P | Pap | 1.5 | III |

[a]Adenoca: adenocarcinoma; M: moderately differentiated; W: well differentiated; P: poorly differentiated; A: anaplastic. [b]Cap: caput pancreatis; Corp: corpus pancreatis; Caud: cauda pancreatis; Pap: papilla Vateri. [c]Largest diameter of the tumour. [d]According to UICC (1987). [e]Dash indicates that no information was available. [f]Diagnosis based on cytologic analysis only.

**Table II** Clonal chromosome aberrations in 13 pancreatic carcinomas

| *Case No.* | *Karyotypes*[a] |
|---|---|
| 2 | 47,XY,+17[2]/46,XY[23] |
| 6 | 45,X,−Y[5]/46,XY[24] |
| 8 | 61-68,XXY,+1,+2,+3,−4,add(4) (p15),+6,+7,der(7)t(7;15)(p22;q11)x2,+der(8)t(8;?;8)(p21;?;q22),add(9)(p13),−11,−12,i(12)(q10),−13,−14,−15,−15,−15,+16,−17,−18,inv(18)(q11q21)x2,−19,der(20)t(?5;20)(q21;p13),−21,+22,der(22)t(8;22)(q11;p11)x2,+2−6mar[10]/46,XY[11] |
| 10 | 46,XY,del(10)(q22q24)[11]/46,XY[4] |
| 11 | 87-93,XX,−X,−X,−1,+2,−3,−4,+5,+i(5)(p10),−6,+7,−9,−10,−10,−12,−13,add(14)(p11),i(15)(q10),+16,+17,−18,+19,+20,+20,−21,+22[16]/160-200,idemx2[7]/46,XX[5] |
| 12 | 40,X,−Y,−3,t(6;18)(q21;q21),−der(8;11)(q10;q10),inv(9)(p11p24),−11,−15,−16,−17,−18,der(19)t(6;19)(q21;p13),+der(22)t(3;22)(q11;p11)[18]/69-87,idemx2[9]/47,XY,+7[4]/46,XY[3] |
| 13 | 42-45,Y,add(X)(p22),−3,i(5)(p10),add(7)(p22),−8,der(9)t(9;12)(p13;q11),del(10)(p11),−12,add(15)(p11),add(16)(q?),der(17)t(1;17)(q21;p13),add(17)(q11),−18,−20,−21,+der(?)t(?;8)(?;q13),+?der(?)t(?;8)(?;q22),+1-2mar[9]/80,idemx2[11]/46,XY[6] |
| 14 | 67-77,XXY,+Y,add(1)(p36),+der(1;16)(q10;p10),+2,del(2)(q33)x2,−3,−4,−5,−6,−7,−8,−9,−10,−10,+11,−12,−13,der(14)t(8;14)(q11;p11)x2,+17,der(17)t(?6;17)(q21;q24)x2,−18,der(19)add(19)(p13)hsr(?),−20,−21,+der(22)t(14;22;?)(q13;q13;?),der(?)t(?;7)(?;q11),+der(?)t(?;7)t(3;7)(p21;q36),+r,+5−13mar[34]/46,XY[2] |
| 16 | 84-86,XXXX,−1,del(3)(p13p24),add(4)(p12-16),dic(5;21)(p15;p11),−6,−9,−12,−13,der(13;15)(q10;q10),add(14)(p11),−15,−18,add(20)(q13),+2-3mar[7]/150-160,idemx2[7]/46,XX[21] |
| 17 | 47,XX,+i(1)(q10)[7]/46,XX[12] |
| 18 | 43-45,XY,−1,der(1)ins(1;?)(p34;?)t(1;1)(p36;p13),add(4)(q35),del(7)(q22q32),−8,+der(11)del(11)(p11)del(11)(q23),i(17)(q10),−18,−22[3]/43-45,idem,+17[3]/43-45,idem,+mar[4]/47,XY,+7[9]/46,XY[4] |
| 19 | 69-79,XXY,+Y,−1,+2,−6,+9,+12,+13[15]/73-74,XXY,+Y,i(1)(q10),+2,−6,+8,+9,−10,+11,+12,+13,+14,+19[13]/78-81,XXY,+Y,+2,+5,−6,+der(7)t(1;7)(q12;q11),+11,+12,+13,+14,−16,+20[15]/150,XY?,inc[5]/46,XY[6] |
| 20 | 46,XY,t(1;19)(p31;q13),t(8;21)(q21;q22)[2]/45,X,−Y[5]/46,XY[10] |

[a]Numbers in square brackets denote number of mitoses in each clone.

two had a single structural change (del(10)(q22q24) in case 10 and +i(1)(q10) in case 17), and the fifth (case 20) had two balanced translocations in one clone and −Y in another. In the remaining eight cases, the tumour karyotype was characterised by numerous numerical and structural aberrations (Figure 1). In two of these tumours (cases 12 and 18) an additional abnormal clone, with +7 as the sole change, was found. The modal chromosome number was neardiploid in eight tumours (hyperdiploid in two, pseudodiploid in two and hypodiploid in four), neartriploid in three, and neartetraploid in two tumours.

Evidence of clonal evolution, i.e., cytogenetically related clones, including duplications and triplications of the mother clone, was found in six tumours (cases 11, 12, 13, 16, 18 and 19). In these polyploid aberrant clones, rings, quadruplications, and other complex chromosome rearrangements were sometimes observed but could, due to the complexity and the high chromosome number in these cells, not always be identified.

The most common numerical changes were (compared with the nearest euploid level) losses of chromosomes 18 (seven cases), 12 (five cases) and 1, 3, 6, 8, 13 and 21 (four cases each). The chromosome bands most frequently involved in structural rearrangements were 1q10, 6q21, 14p11 and 15q10-11 (three cases each) and 1p36, 5p10, 7p22, 7q11, 8q11, 8q22, 9p13, 18q21, 19p13 and 22p11 (two cases each) (Figure 2).

### Discussion

Most of the tumours (17 of 20) of the present series were carcinomas of the exocrine pancreas (Table I), of which 13 (76%) had clonal chromosome aberrations. There seemed to be no cytogenetic difference between the four tumours of the papilla Vateri and the other carcinomas. On the other hand, all three endocrine tumours, two of which were malignant (cases 1 and 5), had normal karyotypes. The numbers are small, but one cannot discount the possiblity that the different findings in the two sets of pancreatic tumours reflect systematic biological differences between neoplasms of the exocrine and endocrine portions of the gland. Of relevance in this context is also that the culture technique we use is more suited to the nutritional necessities and the growth chracteristics of adenocarcinomas, the dominant exocrine pancreatic tumours, than to those of the rare, slowing growing, and biologically highly complex endocrine tumours (Klöppel & Heitz, 1984; Ch'ng *et al.*, 1986). The fact that no previous report has described the cytogenetic investigation of endocrine pancreatic tumours (Mitelman, 1991) adds to the difficulty when it comes to interpreting the importance of the normal karyotypes.

To obtain a more complete picture of the chromosome aberrations in pancreatic adenocarcinomas, we have combined the findings of the present series – 13 tumours with clonal abnormalities – with those of a previous report by us, which included nine pancreatic cancers with clonal chromosome aberrations (Johansson *et al.*, 1992). Our total material therefore consists of 22 karyotypically abnormal pancreatic carcinomas. This represents a significant proportion of the total available data base; we know of only five cytogenetically abnormal cancers of the pancreas reported by other investigators (van der Riet-Fox *et al.*, 1979; Bullerdiek *et al.*, 1985; Casalone *et al.*, 1987; Teyssier, 1987).

The most frequent numerical changes in our combined series were −18 (found in 11 cases, 50%), −Y (found in seven of the 15 male patients with abnormal karyotype, 47%), +20 (eight cases, 36%), +7, +11, and −12 (seven cases each, 32%), and −1, +2, −6, −8, −15, +19 and −21 (five cases each, 23%). Gain of chromosomes 20 and 7 and loss of 18 and Y are among the most common numerical changes also in colorectal adenocarcinomas (Muleris *et al.*, 1990; Bardi *et al.*, 1993), underscoring the karyotypic similarities among the various gastrointestinal cancers. Genetic similarities are evident also at the molecular level, with loss of heterozygosity on 1p, 5q, 11q, 17p and 18q being detected not only in colorectal but also in pancreatic adenocarcinomas (Neuman *et al.*, 1991; Ding *et al.*, 1992; Höhne *et al.*, 1992).

Structural aberrations were present in 17 of the 22 pancreatic cancers with karyotypic abnormalities. Chromosomes 1, 3, 6, 7, 8, 15 and 17 were involved in six or more cases. The most common breakpoint sites were 1p32, 1q10, 6q21, 7p22, 8p21, 8q11, 14p11, 15q10-11, and 17q11 (at least three times, Figure 2). Bands 1q10, 8q11, and 17q11 are also frequently rearranged in colorectal carcinomas (Muleris *et al.*, 1990; Bardi *et al.*, 1993), indicating that genes involved in the initiation or progression of both colorectal and pancreatic malignancies may be located here. The most common karyotypic imbalances (Figure 3) brought about by the structural changes were of chromosomes 1 (both partial and complete losses of 1p and, especially, gains of 1q), 8 (gains of 8q and loss of 8p), and 17 (gain of 17q and loss of 17p). There evidently is no good correspondence between complete and partial polysomies or between complete and partial monosomies.

Cases with simple structural chromosomal rearrangements may be particularly informative about the early genetic changes of cancers, since it can be argued that they must represent primary abnormalities (Mitelman, 1984). This argument can be exemplified by case 17, in which a supernumerary i(1q) was the sole aberration and, hence, must be accepted as a likely candidate for an early and pathogenetically important change. This view is supported by the fact that gain of 1q material was the most frequent imbalance in our series (Figure 3). Isochromosome 1q has, as the only abnormality, otherwise been described in an endometrial carcinoma (Fujita *et al.*, 1985), in an ovarian tumour (Pejovic *et al.*, 1993), and in cancers of the breast (Pandis *et al.*, 1992*b*). As one of several changes, on the other hand, +i(1q) is a frequent finding (Mitelman, 1991).

The diagnosis of case 10, in which a solitary deletion of the long arm of chromosome 10 was found, was mucinous cystadenocarcinoma, a less malignant cancer than ductal adenocarcinoma (Compagno & Oertel, 1979; Hodgkinson *et al.*, 1978). This patient also had nesidioblastosis, a supposedly paraneoplastic disorder of endocrine pancreatic cells that proliferate reactively in close contact with and complicating disease processes of the exocrine pancreas, e.g., pancreatic carcinomas (Eusebi *et al.*, 1981; Klöppel & Heintz, 1984; Permert *et al.*, 1991). Rearrangements of 10q have previously been found in, e.g., thyroid tumours – the only endocrine neoplasms that have been cytogenetically examined in any numbers – and it has been suggested that they characterize nonmedullary carcinomas, especially those whose growth pattern is papillary (Jenkins *et al.*, 1990).

Data are insufficient for any extensive correlation of cytogenetic and pathologic parameters. We nevertheless note that an abnormal karyotype was detected in three of the five well differentiated carcinomas, in nine of the 18 moderately differentiated carcinomas, in seven of the eight poorly differentiated carcinomas, and in both cancers that were classified as anaplastic. A rough parallelism therefore seems to exist between the degree of anaplasia and the likelihood of finding karyotypic abnormalities in cancers of the pancreas.

This work was supported by grants from the Swedish Cancer Society and the Medical Faculty of Lund University. Drs Bardi and Pandis are on leave from the Papanikolaou Research Center, Hellenic Anticancer Institute, Athens, Greece.

### References

ANDRÉN-SANDBERG, Å., AHRÉN, B., TRANSBERG, K.G. & BENGMARK, S. (1991). Surgical treatment of pancreatic cancer. The Swedish experience. *Int. J. Pancreat.*, **9**, 145–152.

BARBACID, M. (1987). Ras genes. *Annu. Rev. Biochem.*, **56**, 779–827.

BARDI, G., JOHANSSON, B., PANDIS, N., BAK-JENSEN, E., ÖRNDAL, C., HEIM, S., MANDAHL, N., ANDRÉN-SANDBERG, Å. & MITTELMAN, F. (1993). Cytogenetic aberrations in colorectal adenocarcinomas and their correlation to clinicopathologic features. *Cancer*, **71**, 306–314.

BARTON, C.M., STADDON, S.L., HUGHES, C.M., HALL, P.A., O'SULLIVAN, C., KLÖPPEL, G., THEIS, B., RUSSELL, R.C.G., NEOPTOLEMOS, J., WILLIAMSON, R.C.N., LANE, D.P. & LEMOINE, N.R. (1991). Abnormalities of the p53 tumour suppressor gene in human pancreatic cancer. *Br. J. Cancer*, **64**, 1076–1082.

BULLERDIEK, J., BARTNITZKE, S., KAHRS, E. & SCHLOOT, W. (1985). Further evidence for nonrandom chromosome changes in carcinoma cells – a report of 28 cases. *Cancer Genet. Cytogenet.*, **16**, 33–43.

CASALONE, R., MERIGGI, F., FORNI, E. & PASQUALI, F. (1987). Cytogenetic findings in a case of anaplastic carcinoma of the pancreas. *Cancer Genet. Cytogenet.*, **29**, 253–259.

CH'NG, J.L.C., POLAK, J.M. & BLOOM, S.R. (1986). Miscellaneous tumors of the pancreas. In *The Exocrine Pancreas: Biology, Pathobiology and Diseases*, Vay Liang, W.G., Gardner, J.D., Brook, F.P. & Ledenthal, E. (eds), pp. 763–771, Raven Press: New York.

COMPAGNO, J. & OERTEL, J.E. (1978). Mucinous cystic neoplasms of the pancreas with overt and latent malignancies (cystadenocarcinoma and cystadenoma). A clinicopathologic study of 41 cases. *Am. J. Clin. Pathol.*, **69**, 573–580.

CUBILLA, A.L. & FITZGERALD, P.J. (1984). Tumors of the exocrine pancreas. In *Atlas of Human Pathology*, Hartman, W.H. & Sobin, L.H. (eds), second series, fascicle 19, Armed Forces Institute of Pathology: Washington, D.C.

DING, S.-F., HABIB, N.A., DELHANTY, J.D.A., BOWLES, L., GRECO, L., WOOD, C., WILLIAMSON, R.C.N. & DOOLEY, J.S. (1992). Loss of heterozygosity on chromosome 1 and 11 in carcinoma of the pancreas. *Br. J. Cancer*, **65**, 809–812.

EUSEBI, V., CAPELLA, C., BOUDI, A., SESSA, F., VEZZADINI, P. & MANCINI, A.M. (1981). Endocrine-paracrine cells in pancreatic exocrine carcinomas. *Histopathology*, **5**, 599–613.

FUJITA, H., WAKE, N., KUTSUZAWA, T., ICHINOE, K., HRESHCHYSHYN, M.M. & SANDBERG, A.A. (1985). Marker chromosomes of the long arm of chromosome 1 in endometrial carcinoma. *Cancer Genet. Cytogenet.*, **18**, 283–289.

GRÜNEWALD, K., LYONS, J., FRÖHLICH, A., FEICHTINGER, H., WEGER, R.A., SCHWAB, G., JANSSEN, W.G. & BARTRAM, C.R. (1989). High frequency of *Ki-ras* codon 12 mutations in pancreatic adenocarcinomas. *Int. J. Cancer*, **43**, 1037–1041.

HEIM, S. & MITELMAN, F. (1992). Cytogenetics of solid tumours. *Recent Adv. Histopathol.*, **15**, 37–66.

HODGKINSON, D.J., REMINE, W.H. & WEILAND, L.H. (1978). A clinicopathologic study of 21 cases of pancreatic cystadenocarcinoma. *Ann. Surg.*, **188**, 679–684.

HÖHNE, M.W., HALATSCH, M.-E., KAHL, G.F. & WEINEL, R.J. (1992). Frequent loss of expression of the potential tumor suppressor gene *DCC* in ductal pancreatic adenocarcinoma. *Cancer Res.*, **52**, 2616–2619.

ISCN (1991). *Guidelines for Cancer Cytogenetics, Supplement to An International System for Human Cytogenetic Nomenclature*, Mitelman, F. (ed.), S. Karger: Basel.

JENKINS, R.B., HAY, I.D. & HERATH, J.F. (1990). Frequent occurrence of cytogenetic abnormalities in sporadic nonmedullary thyroid carcinoma. *Cancer*, **66**, 1213–1220.

JOHANSSON, B., BARDI, G., HEIM, S., MANDAHL, N., MERTENS, F., BAK-JENSEN, E., ANDRÉN-SANDBERG, Å. & MITELMAN, F. (1992). Nonrandom chromosomal rearrangements in pancreatic carcinomas. *Cancer*, **69**, 1674–1681.

KLÖPPEL, G. & HEITZ, P.U. (1984). *Pancreatic Pathology*, pp. 79–113. Churchill Livingstone: Edinburgh.

MARIYAMA, M., KISHI, K., NAKAMURA, K., OBATA, H. & NISHIMURA, S. (1989). Frequency and types of point mutation at the 12th codon of the c-Ki-ras gene found in pancreatic cancers from Japanese patients. *Jpn. J. Cancer Res.*, **80**, 622–626.

MITELMAN, F. (1984). Restricted number of chromosomal regions implicated in aetiology of human cancer and leukaemia. *Nature*, **310**, 325–327.

MITELMAN, F. (1991). *Catalog of Chromosome Aberrations in Cancer*, 4th ed., Wiley-Liss: New York.

MULERIS, M., SALMON, R.-J. & DUTRILLAUX, B. (1990). Cytogenetics of colorectal adenocarcinomas. *Cancer Genet. Cytogenet.*, **46**, 142–156.

NEUMAN, W.L., WASYLYSHYN, M.L., JACOBY, R., ERROI, F., ANGRIMAN, I., MONTAG, A., BRASITUS, T., MICHELASSI, F. & WESTBROOK, C.A. (1991). Evidence for a common molecular pathogenesis in colorectal, gastric and pancreatic cancer. *Genes Chrom. Cancer*, **3**, 468–473.

PANDIS, N., HEIM, S., BARDI, G., LIMON, J., MANDAHL, N. & MITELMAN, F. (1992*a*). Improved technique for short-term culture and cytogenetic analysis of human breast cancer. *Genes Chrom. Cancer*, **5**, 14–20.

PANDIS, N., HEIM, S., BARDI, G., IDVALL, I., MANDAHL, N. & MITELMAN, F. (1992*b*). Whole-arm t(1;16) and i(1q) as sole anomalies identify gain of 1q as a primary chromosomal abnormality in breast cancer. *Genes Chrom. Cancer*, **5**, 235–238.

PEJOVIC, T., HEIM, S., ALM, P., IOSIF, S., HIMMELMANN, A., SKJAERRIS, J. & MITELMAN, F. (1993). Isochromosome 1q as the sole karyotypic abnormality in a Sertoli cell tumor of the ovary. *Cancer Genet. Cytogenet.* (in press).

PERMERT, J., MOGAKI, M., ANDRÉN-SANDBERG, Å., KAZAKOFF, K. & POUR, P.M. (1991). Pancreatic mixed ductal-islet tumors. Is this an entity? *Int. J. Pancreat.*, **11**, 23–29.

SHIBATA, D., ALMOGUERA, C., FORRESTER, K., DUNITZ, J., MARTIN, S.E., COSGROVE, M.M., PERUCHO, M. & ARNHEIM, N. (1990). Detection of c-K-ras mutations in fine needle aspirates from human pancreatic adenocarcinomas. *Cancer Res.*, **50**, 1279–1283.

TEYSSIER, J.R. (1987). Nonrandom chromosomal changes in human solid tumors: application of an improved culture method. *J. Natl Cancer Inst.*, **79**, 1189–1198.

UICC (1987). *TNM Classification of Malignant Tumors*, Hermanek, P. and Sobin, L.H. (eds), 4th ed., Springer-Verlag: Berlin.

VAN DER RIET-FOX, M.F., RETIEF, A.E. & VAN NIEKERK, W.A. (1979). Chromosome changes in 17 human neoplasms studied with banding. *Cancer*, **44**, 2108–2119.